# Comparison of Hamilton Depression Rating Scale and Montgomery-Åsberg Depression Rating Scale: Baked Straight From a Randomized Study

**DOI:** 10.7759/cureus.45098

**Published:** 2023-09-12

**Authors:** N Simple Santi, Sashi B Biswal, Birendra Narayan Naik, Jyoti Prakash Sahoo, Bhabagrahi Rath

**Affiliations:** 1 Pharmacology, VIMSAR (Veer Surendra Sai Institute of Medical Sciences and Research), Burla, IND; 2 Psychiatry, VIMSAR (Veer Surendra Sai Institute of Medical Sciences and Research), Burla, IND; 3 Pharmacology, Kalinga Institute of Medical Sciences, Bhubaneswar, IND

**Keywords:** correlation analysis, bland-altman plot, escitalopram, vortioxetine, vilazodone, serotonin receptor, beck’s depression inventory, montgomery-asberg depression rating scale, hamilton depression rating scale, depressive disorder

## Abstract

Background and objectives: The symptoms of major depressive disorder (MDD) are nowadays being assessed with the Hamilton and Montgomery-Åsberg Depression Rating Scales. However, there are few studies on the comparison of these two scales. Our study aimed to determine the correlation between the Hamilton Depression Rating Scale (HDRS) and Montgomery-Åsberg Depression Rating Scale (MADRS) scores at baseline through 12 weeks.

Methods: An ongoing randomized, open-label, three-arm study's interim analysis is portrayed here. The participants were assessed with HDRS and MADRS at baseline, four, eight, and 12 weeks after receiving oral tablets of either vilazodone (20-40 mg/d), escitalopram (10-20 mg/d), or vortioxetine (5-20 mg/d). This study is prospectively registered with the Clinical Trial Registry, India (CTRI/2022/07/043808).

Results: Of 71 recruited individuals, 49 (69%) completed the 12-week visit. At baseline, the three groups' median HDRS scores were 30.0, 29.5, and 29.0 (p=0.76), and at 12 weeks, they reduced to 19.5, 19.5, and 18.0 (p=0.18). At baseline, the group-wise median MADRS scores were 36, 36, and 36 (p=0.79); at 12 weeks, they were 24, 24, and 23 (p=0.03). The Pearson correlation revealed that the association between the changes in scores from baseline was strongest for escitalopram (r=0.70, p=0.002) followed by vortioxetine (r=0.59, p=0.01) and vilazodone (r=0.59, p=0.02). The Bland-Altman analysis showed that the mean difference between the scores was 5.11 (95% CI: 3.08-7.14).

Conclusion: According to this interim study, HDRS and MADRS scores declined after 12 weeks of therapy. Both scores had strong positive correlation, and the difference between the scores reduced with time.

## Introduction

The global incidence of major depressive disorder (MDD) has fostered a sharp rise in the past two decades [1]. According to recent statistics, the annual prevalence of MDD is 15.9% in India [2]. The Hamilton Depression Rating Scale (HDRS) [3] is the most widely utilized tool for assessing depressive symptoms for diagnosis and monitoring of therapy. The Montgomery-Åsberg Depression Rating Scale (MADRS) [4] is also routinely leveraged to assess treatment outcomes of antidepressant medications. According to both the scales, i.e., HDRS and MADRS, the depressive disorder is categorized into major or severe, moderate, mild, borderline or minimal depression, and no depression. In addition to these two objective scales, the Beck Depression Inventory (BDI) [5] is the most accepted subjective scale for evaluating depressive symptoms. It has not been assessed precisely how the HDRS and MADRS function to measure symptom ratings in individuals with depressive disorder [6].

It is unclear which antidepressant substantially minimizes the symptoms of depression, although there are several potent antidepressants available on the market. Furthermore, there is a lack of information regarding the magnitude and sustainability of such impacts [7]. Based on the literature survey, we planned this study with three antidepressant drugs, namely, escitalopram, a selective serotonin receptor inhibitor (SSRI); vilazodone, an SSRI with partial agonistic action at 5-HT1A receptors; and vortioxetine, a serotonin modulator plus transporter inhibitor [8-10]. The hypothesis that newer antidepressants might alleviate MDD patients' symptoms underpinned this study. Some previous studies [6,11-14] also compared the HDRS and MADRS tools. This study aimed to determine the correlations between the HDRS and MADRS scores at 12 weeks, their changes from baseline, and the comparison of both scales using the Bland-Altman analysis [15].

## Materials and methods

This analysis within a randomized, open-label, three-arm study weighs the HDRS and MADRS scores of those with MDD who received either vilazodone, escitalopram, or vortioxetine for 12 weeks. We started enrolling patients in the Department of Psychiatry, VIMSAR (Veer Surendra Sai Institute of Medical Sciences and Research), Burla, India, in July 2022. Before group assignment, participants or their family members provided written informed consent for the research program. The Institutional Ethics Committee, VIMSAR, Burla, Odisha, India, granted us ethical approval before the study's commencement (No. 029-2022/I-S-T/03, dated: May 17, 2022). Our study is prospectively registered with the Clinical Trial Registry, India (CTRI/2022/07/043808). The study complied with the Declaration of Helsinki, the Good Clinical Practice Guidelines of the International Council for Harmonization, and other institutional standards.

Study participants

With an HDRS score of below 24, individuals with MDD between 18 and 65 years old were included. Patients with neurodegenerative conditions, psychotic symptoms, kidney disease (baseline estimated glomerular filtration rate < 45 ml/min/1.73 m^2^), cardiovascular events within the past six months, serum alanine transaminase (ALT) or aspartate transaminase (AST) levels > 150% of the standard limit, serum triglyceride > 400 mg/dl, and pregnant or lactating women were excluded from the study. Participants are permitted to withdraw their consent at any time without justification.

Study design and endpoints

The participants were randomly assigned to one of the following drug groups in a 1:1:1 ratio: group A received vilazodone tablets 20-40 mg once daily, group B received escitalopram tablets 10-20 mg once daily, and group C received vortioxetine tablets 5-20 mg once daily. This was done by using permuted block randomization using blocks 12 and 24. We stratified the randomization procedure based on gender (female or male) and status of depression (not receiving therapy or using antidepressants for < 6 months).

The primary objectives for this interim analysis were the correlations between the HDRS and MADRS scores at baseline, week 12, and their changes from baseline. A secondary objective was the comparison of the HDRS and MADRS scores from the baseline visit through week 12 using the Bland-Altman analysis [15]. On the per-protocol (PP) population, the analyses were focused.

Study procedure

Each participant received one of the following antidepressant tablets for the entire study duration: vilazodone 20-40 mg once daily (group A), escitalopram 10-20 mg once daily (group B), or vortioxetine 5-20 mg once daily (group C). These drugs were provided to the participants freely by the principal investigator. The psychiatrist adjusted the dosage based on the participant's clinical outcome. Cross-over was not permitted. Participants were thoroughly evaluated for their psychological and physical health at the first session. Following the baseline visit, we scheduled follow-up visits to assess the HDRS and MADRS scores at four, eight, and 12 weeks. We correlated the individual scores and their changes from baseline with the Pearson correlation. We also performed the Bland-Altman analysis to compare the two scales mentioned above.

Statistical analysis

The sample size was finalized for the primary endpoint of the larger ongoing study, i.e., change in HDRS score after 16 weeks. With a mean reduction in HDRS of 10.0 from baseline and a standard deviation of 2.0, 87 patients (29 in each group) were required to quantify a change in HDRS with 80% power at a 0.05 two-sided significance level. For this study, 96 participants (32 in each group) were chosen after accounting for an attrition rate of 10%. We conducted an interim analysis following the first 48 individuals' 12-week visit.

The Shapiro-Wilk test was used to confirm the data's normality. The continuous data was shown with the median and interquartile range (IQR). The Kruskal-Wallis test was applied to analyze them followed by post hoc analysis by the Bonferroni test. The categorical data were shown as the frequency with proportion, and Pearson's chi-square (ꭓ^2^) test was used for their evaluation. For data analysis and generation of plots, we used the R software (version 4.2.3) [16]. Each statistical test was two-tailed, and a p-value < 0.05 was considered statistically significant.

## Results

**Figure 1:**
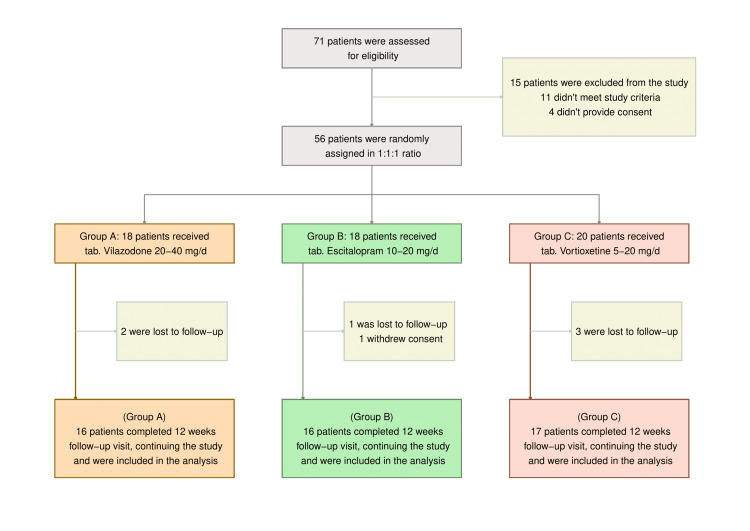
The CONSORT diagram The CONSORT (Consolidated Standards of Reporting Trials) diagram shows the screening, randomization, and completion of study visits till the interim analysis. Eleven patients were ineligible according to the study criteria. Ten were beyond 18-65, and one was 12 weeks pregnant at the baseline visit. This interim analysis was done in the per-protocol (PP) population (n = 49).

Seventy-one patients were screened to ascertain eligibility. One pregnant lady, four people reluctant to consent, and 10 additional respondents beyond the required age limit were excluded from the study. To one of the three study groups, each of the other 56 participants was randomized. Six other participants (two, one, and three in the relevant study groups) skipped their follow-up appointments, and one person in the escitalopram group withdrew her consent. In this interim analysis, 49 subjects (24 females and 25 males; 16 in the vilazodone, 16 in the escitalopram, and 17 in the vortioxetine groups) underwent evaluation (Figure 1). Participants in all three study groups possessed similar baseline features (Table 1).

**Table 1:** Baseline characteristics of the study population (n = 49) The continuous variables were expressed as the median (interquartile range). The categorical values were presented as n (%). BMI: body mass index; HDRS: Hamilton Depression Rating Scale-17 items version; MADRS: Montgomery-Åsberg Depression Rating Scale.

Parameters	Total (n = 49)	Group A Vilazodone (n = 16)	Group B Escitalopram (n = 16)	Group C Vortioxetine (n = 17)	p-Value
Age (years)	44.0 (34.0-55.0)	47.5 (38.8-53.5)	42.0 (33.0-54.5)	43.0 (34.0-55.0)	0.094
Gender					
Female	25 (51.0%)	8 (50.0%)	8 (50.0%)	9 (52.9%)	0.981
Male	24 (49.0%)	8 (50.0%)	8 (50.0%)	8 (47.1%)	
BMI (kg/m^2^)	27.1 (25.6-28.5)	26.6 (25.4-28.5)	26.9 (26.3-27.8)	28.0 (26.0-28.6)	0.126
HDRS	30.0 (29.0-31.0)	30.0 (29.0-31.0)	29.5 (29.0-30.0)	29.0 (29.0-32.0)	0.763
MADRS	36.00 (35.00-37.00)	36.00 (35.00-37.00)	36.00 (35.00-36.25)	36.00 (35.00-38.00)	0.789

**Figure 2:**
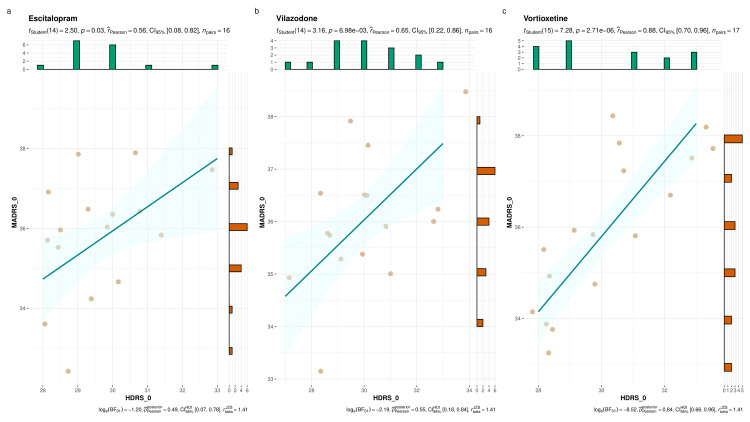
Baseline HDRS and MADRS scores of the study participants The plots a, b, and c illustrate the correlation of baseline HDRS and MADRS scores of study participants from escitalopram, vilazodone, and vortioxetine groups, respectively. The jitter plots denote individual participants with their corresponding scores. The histograms on the X and Y axes represent the number of participants with their baseline HDRS and MADRS scores. The Pearson correlation was performed. HDRS_0: Baseline score according to Hamilton Depression Rating Scale; MADRS_0: Baseline score according to Montgomery-Åsberg Depression Rating Scale.

Figure 2 showcases the baseline HDRS and MADRS scores of the study participants. The three parts (i.e., Figures 2a-c) illustrate the scores of escitalopram, vilazodone, and vortioxetine groups, respectively. We performed the Pearson correlation and found positive correlation between both scores. However, the strength of association was most significant for the vortioxetine group (r=0.88, p<0.001), followed by the vilazodone group (r=0.65, p=0.007), and the escitalopram group (r=0.56, p=0.03).

**Figure 3:**
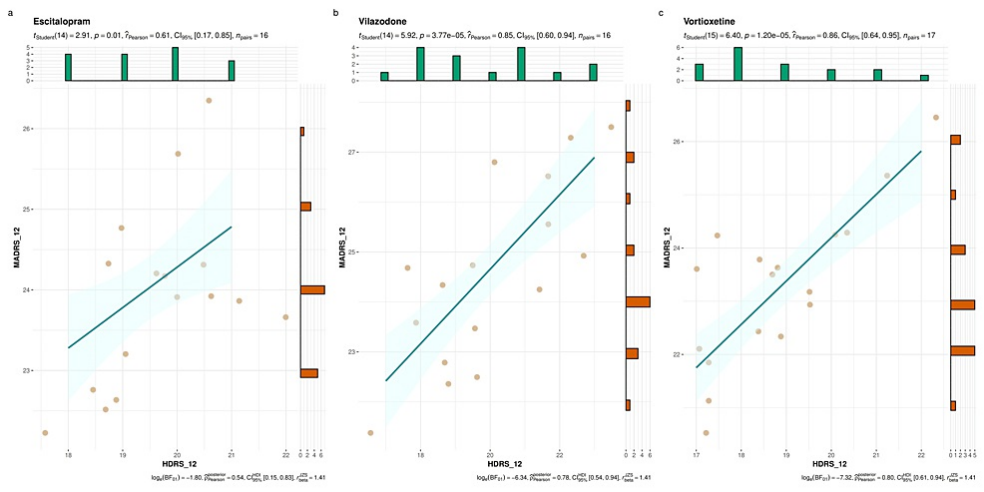
HDRS and MADRS scores of the study participants at 12 weeks The plots a, b, and c illustrate the correlation of HDRS and MADRS scores of study participants from escitalopram, vilazodone, and vortioxetine groups, respectively, after 12 weeks of the interventions. The jitter plots denote individual participants with their corresponding scores. The histograms on the X and Y axes represent the number of participants with their HDRS and MADRS scores at 12 weeks. The Pearson correlation was performed. HDRS_12: Score at 12 weeks according to Hamilton Depression Rating Scale; MADRS_12: Score at 12 weeks according to Montgomery-Åsberg Depression Rating Scale.

Figure 3 highlights the HDRS and MADRS scores of the study participants at 12 weeks. The three parts (i.e., Figures 3a-c) illustrate the scores of escitalopram, vilazodone, and vortioxetine groups, respectively. We found positive correlation between both scores. However, the strength of association was maximum for the vortioxetine group (r=0.86, p<0.001), followed by the vilazodone group (r=0.85, p<0.001), and the escitalopram group (r=0.61, p=0.01).

**Figure 4:**
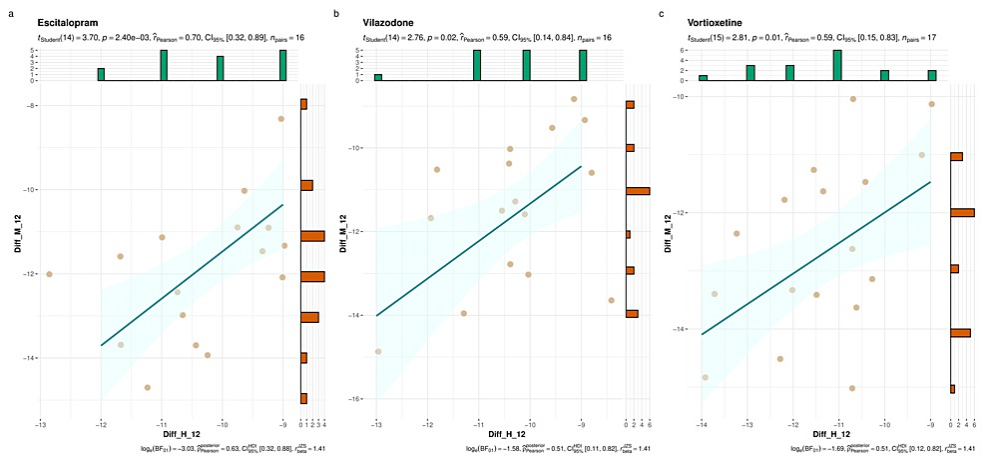
Changes in HDRS and MADRS scores from baseline The plots a, b, and c illustrate the correlation of changes in HDRS and MADRS scores of study participants from escitalopram, vilazodone, and vortioxetine groups, respectively at 12 weeks from their baseline values. The jitter plots denote individual participants with their corresponding scores. The histograms on the X and Y axes represent the number of participants with their difference in the HDRS and MADRS scores. The Pearson correlation was performed. Diff_H_12: Change in Hamilton Depression Rating Scale at week 12 from baseline; Diff_M_12: Change in Montgomery-Åsberg Depression Rating Scale at week 12 from baseline.

Figure 4 displays the changes in the participants’ HDRS and MADRS scores from their baseline values. The three parts (i.e., Figures 4a-c) illustrate the scores of escitalopram, vilazodone, and vortioxetine groups, respectively. Using the Pearson correlation, we found strong positive correlations between both scores. However, the strength of association was highest for the escitalopram group (r=0.70, p=0.002), followed by the vortioxetine group (r=0.59, p=0.01), and the vilazodone group (r=0.59, p=0.02).

**Figure 5:**
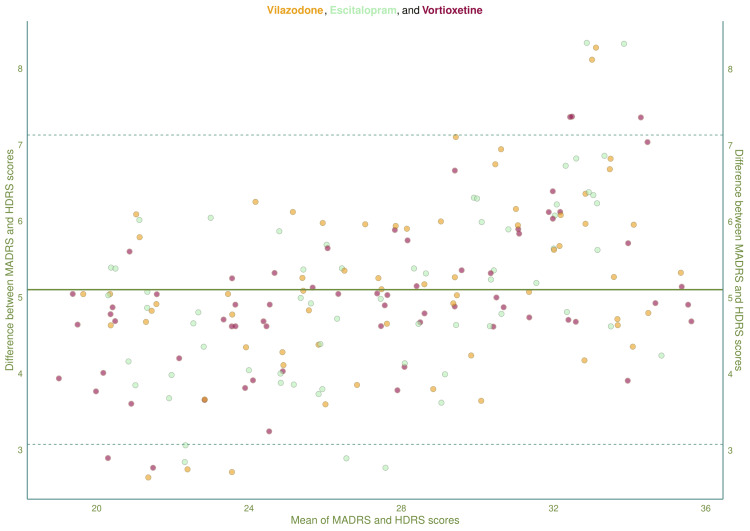
Bland-Altman plot for comparison of the two scales (HDRS and MADRS) The Bland-Altman plot illustrates the comparison of HDRS and MADRS scores of all study participants at the baseline through week 12. The X and Y axes represent the mean and differences of both scores. The solid and dashed lines represent the mean difference and confidence intervals. HDRS: Hamilton Depression Rating Scale-17 items version; MADRS: Montgomery-Åsberg Depression Rating Scale.

Figure 5 shows the Bland-Altman plot, which compares the means and differences of the HDRS and MADRS scores of the study population. The mean difference between the scores was 5.11 (95% CI: 3.08-7.14). With the advancement of study duration, the individual scores, along with the mean scores, reduce. The difference between the scores narrows down with the reduction of mean scores. It implies that the HDRS and MADRS scores can be utilized interchangeably for milder cases of depressive disorders.

## Discussion

The three study drugs significantly lowered the HDRS and MADRS scores at 12 weeks, judging by this ongoing study's interim analysis. A strong positive association was evident between the HDRS and MADRS scores. After we analyzed the two scores, our results and those from the meta-analysis by Heo et al. [6] matched.

Daily dosages for the experimental groups were 20-40 mg of vilazodone and 5-20 mg of vortioxetine, while for the control group, it was 10-20 mg of escitalopram. Vilazodone has the perk of being a partial agonist at the 5-HT1A receptor, while the control, escitalopram, only has one mode of action. However, vortioxetine impedes serotonin receptors and restricts its transport. In this study, we found that the HDRS tool was useful in the diagnosis of MDD and the MADRS tool was very effective during the assessments of treatment outcomes. The HDRS focuses almost equally on somatic and psychological symptoms. On the other hand, MADRS focuses more on the psychological than the somatic symptoms. However, both of these scores do not address uncommon symptoms like hypersomnia and hyperphagia. The scattered plots and the Bland-Altman plot collectively depict that the two scales used in this study are positively associated and have substantial similarities. We have also discovered that the vortioxetine group's reduction in HDRS and MADRS scores, improvements in quality of life, and metabolic parameters were significantly higher than those of the other two groups [17-19]. These findings imply that vortioxetine monotherapy could be effective in managing MDD.

All people in the study got free medication, irrespective of their allocation. This led to a low attrition rate. After 12 weeks, all study participants' HDRS and MADRS scores decreased substantially. The strong positive linear association (r=0.6-0.9, p<0.05) between the HDRS and MADRS scores prevailed throughout the study, including even the difference from baseline values. As per the study by Reijnders et al. [12], the HDRS and MADRS tools can be interchangeably used to diagnose depressive disorder. Our results aligned with their findings. According to a meta-analysis by Heo et al. [6], the HDRS and MADRS are equivalent while assessing the severity of symptoms in depression. This study explores the association of the scores. The results should be deemed preliminary, as this was the initial assessment of a more extensive study. Further analyses are needed to establish whether the drugs have a long-lasting effect on the HDRS and MADRS scores.

The Bland-Altman analysis [15] was the core strength of this comparative study. Additional strengths were routine follow-up visits and weighing depressive symptoms with widely used HDRS and MADRS. Our study had a few things that could have been improved. First, the open-label study design might have contributed to the dropouts. Second, the medications were provided free of charge. The study results show that the high price of the study drugs could constrain practical applicability. Third, the above conclusions are based on an interim examination of a more extensive, ongoing study. Establishing the consistency between these findings and those of the ongoing, more extensive study is essential.

## Conclusions

According to this interim analysis, after completing the therapeutic intervention for 12 weeks, each participant's HDRS and MADRS scores significantly decreased. Both scores exhibited a strong positive correlation, and their differences were reduced over time. Further analysis of such effects is required.
